# A review of research progress on mechanisms of peritoneal fibrosis related to peritoneal dialysis

**DOI:** 10.3389/fphys.2023.1220450

**Published:** 2023-09-25

**Authors:** Jin’e Li, Yinghong Liu, Jianping Liu

**Affiliations:** ^1^ Department of Endocrinology and Metabolism, The Second Affiliated Hospital of Nanchang University, Nanchang, China; ^2^ Jiangxi Medical College, Nanchang University, Nanchang, China; ^3^ Department of Nephrology, Second Xiangya Hospital, Central South University, Changsha, China

**Keywords:** peritoneal dialysis, peritoneal fibrosis, EMT, TGF-β1, mechanism

## Abstract

Peritoneal dialysis (PD) is an effective alternative treatment for patients with end-stage renal disease (ESRD) and is increasingly being adopted and promoted worldwide. However, as the duration of peritoneal dialysis extends, it can expose problems with dialysis inadequacy and ultrafiltration failure. The exact mechanism and aetiology of ultrafiltration failure have been of great concern, with triggers such as biological incompatibility of peritoneal dialysis solutions, uraemia toxins, and recurrent intraperitoneal inflammation initiating multiple pathways that regulate the release of various cytokines, promote the transcription of fibrosis-related genes, and deposit extracellular matrix. As a result, peritoneal fibrosis occurs. Exploring the pathogenic factors and molecular mechanisms can help us prevent peritoneal fibrosis and prolong the duration of Peritoneal dialysis.

## 1 Introduction

The peritoneum is made up of a single layer of mesoderm-derived mesothelial cells and a thin layer of connective tissue consisting of a dense subepithelial band. The dense band, composed mainly of collagen fibre bundles, along with some lymphocyte tubes, fibroblasts, mast cells, macrophages, and capillaries, plays a crucial role in maintaining the function and structure of the peritoneum (Bajo et al., 2017). The peritoneal natural semipermeable membrane is responsible for ultrafiltration and solute diffusion during dialysis by converting solutes and water and removing metabolites while maintaining water and electrolyte balance.

Peritoneal dialysis is a cost-effective method of dialysis (Li et al., 2017) that is gaining international attention. However, various nonphysiological factors in the dialysate, such as hyperosmolarity, hyperglycaemia, low pH, glucose degradation products (GDPs), and advanced glycosylation end products (AGEs), can lead to chronic stimulation and damage of the peritoneum in PD patients, causing peritoneal sclerosis. Peritoneal fibrosis has always been a hot topic of research. Here, we provide an overview of how factors such as inflammation, oxidative stress, glucose metabolism, and hypoxia mediate peritoneal fibrosis through cytokine generation and molecular pathway activation.

## 2 EMT induced by TGF-β

Epithelial-mesenchymal transition (EMT) is a complex biological process characterized by the gradual loss of epithelial-specific markers, such as E-cadherin and zonula occludens-1, and the acquisition of a fibroblast phenotype expressing fibroblast-specific protein 1 (FSP1) and α-smooth muscle actin (α-SMA). This process is accompanied by changes in cellular behaviour and the production of extracellular matrix (ECM) (Kalluri and Neilson, 2003).

**FIGURE 1:**
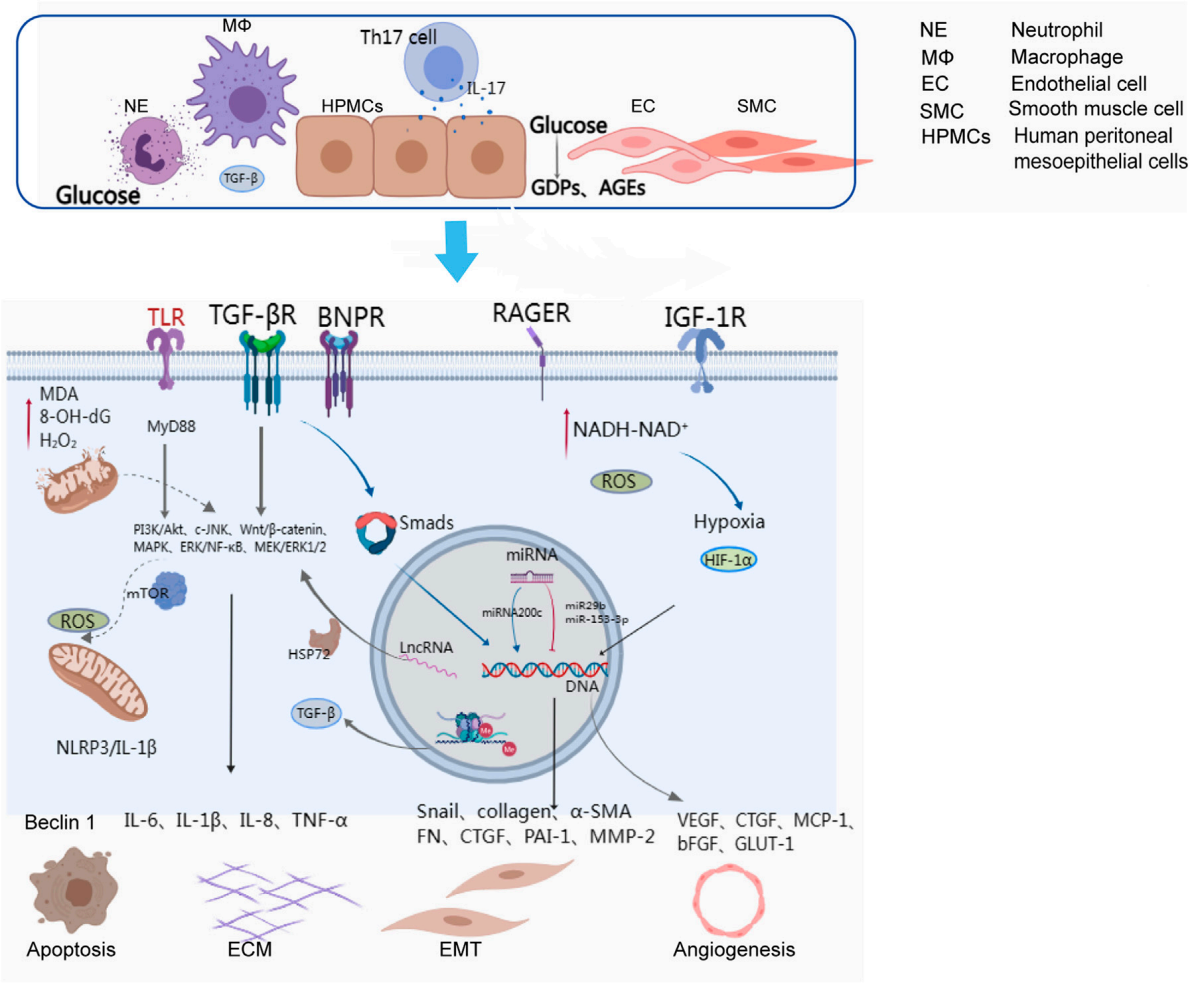
Cells and activated molecular pathways involved in peritoneal ultrafiltration failure.

The exact mechanisms underlying the EMT of peritoneal mesothelial cells remain unclear, but they are thought to involve the interaction of cytokines, inflammatory factors, and transcription regulators. Evidence suggests that Smad and non-Smad signalling pathways induced by TGF-β1 play a dominant role in the EMT of peritoneal fibrosis (Lho et al., 2021). In the early phase of fibrosis, glucose, GDPs, and advanced glycosylation end products (AGEs) can upregulate type I and type II TGF-β receptors in mesothelial cells (Suryantoro et al., 2023) by activating protein kinase C-α (PKC-α) (Wang et al., 2016). TGF-β1 signalling activates the phosphorylation of Smad2 and Smad3 via type I TGF-β receptors, and Smad2/Smad3 are transported to the nucleus, where they directly bind to DNA and regulate the transcription of target genes, including Snail, collagen, α-SMA, fibronectin, CTGF, β-catenin, plasminogen activator inhibitor-1 (PAI-1), and matrix metalloproteinase-2 (MMP2), promoting fibrosis (Derynck and Zhang, 2003; Hirahara et al., 2009; Xiao et al., 2010; Lei et al., 2012; Zhang et al., 2022a; Huang et al., 2022; Masola et al., 2022) (Figure 1). Smad1/5/8 proteins activated by ALKs in response to BMP (bone morphogenetic proteins) one to four or other ligands are also transported to the nucleus to regulate the transcription of target genes (Balzer, 2020). In addition to the Smad-dependent signalling pathway, there are also various non-Smad signalling pathways involved in the process of fibrosis, such as PI3K/Akt, c-Jun N-terminal kinases (JNK), Wnt/β-catenin, and ERK/NF-κB (Liu et al., 2012; Jang et al., 2013; Zhang et al., 2013; Lamouille et al., 2014).

Numerous studies have reported various initiating factors in epithelial-mesenchymal transition (EMT). For instance, caveolin-1 has been proposed to play a critical role in EMT associated with peritoneal fibrosis in patients with Parkinson’s disease (Strippoli et al., 2015). Furthermore, overexpression of insulin-like growth factor 1 receptor (IGF-1R) has been linked to the promotion of EMT in human peritoneal mesothelial cells (Xia et al., 2022). These findings have expanded our understanding of potential strategies for preventing and treating EMT.

## 3 Inflammation

Inflammation is a frequent underlying cause of peritoneal fibrosis in patients with PD. While it can be directly induced by pathogenic microorganisms, peritoneal inflammation may also result from the accumulation of uraemia toxins, mechanical stress on blood vessel walls, ageing, and complications of diabetes (Cueto-Manzano et al., 2007). In PD patients, an increase in the number of peritoneal macrophages suggests chronic peritoneal inflammation, even in the absence of acute peritonitis.

The inflammatory response is a complex process involving multiple molecular mechanisms, inflammatory mediators, and signalling pathways. The peritoneum can be damaged by various factors, activating macrophages and neutrophils that mediate NF-κB signalling pathways via Toll-like receptors (TLRs) and release numerous inflammatory cytokines, such as IL-6, IL-1β, IL-8, and TNF-α, leading to peritoneal fibrosis (Kato et al., 2004). TLRs can also activate the downstream molecules of JNK, p38MAPKs, and ERK1/2 through the MyD88 signalling pathway, promoting the expression of proinflammatory cytokines. In addition, the NLRP3 inflammasome, a cellular complex composed of proteins, can activate the cysteine-dependent aspartate-directed proteolytic enzyme and induce the production of IL-1 and IL-18, resulting in inflammatory responses (Hautem et al., 2017) (Figure 1). IL-6 plays a crucial role in regulating inflammation and can be secreted by macrophages, monocytes, and human peritoneal mesothelial cells. Exposure to dialysate and IL-1β can trigger the release of IL-6, which has been reported to increase proportionally with the glucose concentration in the dialysate (Milan Manani et al., 2016; Yang et al., 2020). IL-6, along with soluble IL-6 receptors, can facilitate the synthesis and secretion of MCP-1, MCP-3, and IL-8, as well as adhesion molecules and angiogenesis factors (Salgado et al., 2002; Bellón et al., 2011). IL-17, mainly produced by Th17 cells and γδ T cells, was detected in peritoneal biopsies of PD patients but not healthy subjects (Rodrigues-Díez et al., 2014). IL-17 can promote the production of ELR + CXC chemokines in mesothelial cells, such as CXCL1 and CXCL8. Furthermore, it is believed that IL-17 might directly stimulate mesothelial cells to produce VEGF via an unidentified mechanism (Witowski et al., 2018).

In addition, recent studies have begun to focus on the cyclic GMP-AMP synthase (cGAS)-stimulator of interferon genes (STING) pathway (Decout et al., 2021; Zhang et al., 2022b). Inflammation leads to damage to mitochondrial DNA (mtDNA), which can be released into the cytoplasm under cellular stress and recognized by various DNA sensing mechanisms, including TLR-9, cytoplasmic cGAS-STING signalling, and inflammasome activation, which can result in the development of fibrosis, as well as pathological angiogenesis and endothelial-to-mesenchymal transition (Wang et al., 2022a; Wang et al., 2022b). In the future, there may be more research on peritoneal fibrosis related to peritoneal dialysis that focuses on the cGAS-STING pathway.

## 4 Regulation of epigenetic genes

The role of epigenetic modifications in fibrosis has garnered increasing recognition. Epigenetic regulation mainly includes DNA methylation, histone modification, and noncoding RNA regulation. It is widely believed that DNA methylation of cytosine-phosphate-guanine (CpG) dinucleotide sites near the gene promoter leads to target gene silencing. In 2017, Kim et al. found that DNMT1 (DNA methyltransferase 1) inhibits RASAL1 protein expression by promoting hypermethylation of RASAL1 (Ras GTPase activating-like protein 1), which in turn upregulates the expression of TGF-β1 and accelerates peritoneal thickening *in vivo* in experiments with encapsulating peritoneal sclerosis rats (Kim et al., 2014). Histone methylation has also been implicated in PF, as Maeda et al. demonstrated that the expression of H3K9 histone methyltransferase and H3K4 methyltransferase can exacerbate the thickening of the submesothelial compact zone (Maeda et al., 2017). Recent studies have also linked high expression of EZH2 (Zeste homologue enhancer 2) with peritoneal fibrosis, possibly through the activation of profibrotic signalling pathways (Wang et al., 2020; Shi et al., 2022).

Furthermore, there is a growing body of evidence indicating that different types of noncoding RNAs, including microRNAs (miRNAs) and long-chain noncoding RNAs (lncRNAs), are involved in the transcription of genes that regulate peritoneal fibrosis (Liu et al., 2019) (Figure 1). Studies have suggested that certain miRNAs, such as miR-129–5p and miRNA-302c, have a protective effect on the peritoneum during dialysis, while others, such as miRNA-30b, miRNA-23, and miRNA-21, can exacerbate peritoneal fibrosis (Xiao et al., 2015; Morishita et al., 2016a; Hirai et al., 2017; Lopez-Anton et al., 2017; Yanai et al., 2018; Zhang et al., 2019). The functions of miRNAs are diverse and complex, primarily involving the regulation of gene expression through interactions with DNA, RNA, proteins, or their combinatorial interactions involved in transcriptional and posttranscriptional regulation (Guo et al., 2020). Some researchers also believe that lncRNAs, such as BC049991 and AK080622, may participate in peritoneal fibrosis by influencing heat shock protein 72 (HSP72) (Bergmann and Spector, 2014). Additionally, lncRNA AV310809 promotes TGF-β1 by activating the Wnt2/β-catenin signalling pathway, which induces EMT in human peritoneal mesothelial cells (Bergmann and Spector, 2014). While circular RNAs (circRNAs) have been implicated in organ fibrosis, such as cardiac fibrosis, liver fibrosis, and pulmonary fibrosis (Li et al., 2019; Yousefi and Soltani, 2021), relatively little is known about their involvement in peritoneal fibrosis. It is possible that circRNAs may be involved in peritoneal fibrosis by inducing EMT, but further research is needed to determine the specific principles and molecular mechanisms.

## 5 Autophagy and apoptosis

Autophagy is a double-edged sword. On the one hand, it can be activated by cells to prevent organelle damage caused by reactive oxygen species (ROS) under certain external stimuli. However, it may also induce epithelial apoptosis or EMT. Studies have shown that autophagy initiation can prevent peritoneal tissue damage by blocking NLRP3/IL-1β-mediated inflammasome activation (Wu et al., 2016) (Figure 1).

Normally, autophagy activation and inhibition are in balance, and excessive activation or inhibition can lead to oxidative stress, inflammatory damage, and even fibrosis. In fact, it has been reported that rapamycin, an autophagy inducer, stimulates mTOR signalling in peritoneal mesodermal cells activated by hyperglycaemia, reducing EMT and improving peritoneal fibrosis (Wu et al., 2018). Conversely, high glucose peritoneal dialysate has been shown to induce autophagy and fibrosis in human peritoneal mesothelial cells (Wu et al., 2018). This may be involved in EMT, the proinflammatory response, and angiogenesis by regulating the TGF-β/Smad3, EGFR/ERK1/2, STAT3/NF-κB, and β-catenin axes (Shi et al., 2021), suggesting that autophagy plays a role in peritoneal fibrosis.

Autophagy is reported to contribute to apoptosis as type II programmed cell death via autodigestive cellular progression or extracellular stimulation (Ghavami et al., 2015; Zou et al., 2016). Some researchers have reported that high-glucose peritoneal dialysate induces Beclin 1-dependent autophagy in human peritoneal mesoepithelial cells (HPMCs) and that autophagy inhibition reduces EMT, fibrosis and apoptosis in HPMCs (Wu et al., 2018). It has also been suggested that promoting mitochondrial synthesis and inhibiting apoptosis can improve peritoneal fibrosis (Li et al., 2022).

## 6 Pseudohypoxia and angiogenesis

Angiogenesis is considered to be a significant factor in peritoneal ultrafiltration failure (Schilte et al., 2009). Neovascularization increases the effective surface area for solute exchange and decreases osmotic pressure driven by glucose from the peritoneal dialysate, resulting in a decrease in ultrafiltration volume. Neovascularization is often associated with hypoxia in physiological or pathological states (Lei et al., 2012). Peritoneal mesenchymal cells overmetabolize glucose, leading to pseudohypoxia with increased NADH-NAD+ (oxidized-reduced nicotinamide dinucleotide ratio) in the cytosol (Krediet and Parikova, 2022) (Figure 1). This ratio promotes the expression of hypoxia-inducible factor-1 (HIF-1), which is one of the most important regulators in the hypoxia response. HIF-1 can regulate the transcription of various genes, mediate the occurrence of EMT, and thus participate in peritoneal fibrosis. YANG et al. have suggested that the activation of the HIF-1α/STAT3 signaling pathway is the main contributor to EMT of mesenchymal cells induced by high glucose, and that knockdown of HIF-1α could alleviate the EMT and fibrosis process (Yang et al., 2021). Yoshiyuki et al. found that hypoxia can promote the expression of HIF-1α, Snail-1, VEGF, and MMP-2 in rat mesenchymal cells, which induces EMT, and that HIF-1α inhibitors can diminish fibrosis by suppressing the expression of these factors (Morishita et al., 2016b). VEGF is a key player in peritoneal angiogenesis, and studies have shown that peritoneal dialysis solution with glucose is related to the growth of VEGF (Zweers et al., 2001). GDPs in PD solution can induce the formation of AGEs, and the AGE (RAGE) receptor plays a profibrotic role by mediating the activation of VEGF to induce capillary angiogenesis and promoting TGF-β-induced EMT (De Vriese et al., 2006; Boulanger et al., 2007). Angiogenesis is regulated by cell growth factors such as VEGF and basic fibroblast growth factor (bFGF), as well as statins such as angiostatin and endostatin (ES) (Kakuta et al., 2005; Tanabe et al., 2007; Nakao et al., 2010).

Apart from VEGF, several other factors contribute to peritoneal neovascularization. Aquaporin-1 (AQP 1), which is responsible for fluid transport, plays a vital role in angiogenesis and endothelial cell migration (Saadoun et al., 2005). Angiotensin 2 (Ang-2) is an angiogenic factor that has been found to be involved in peritoneal neovascularization in rats with EPS (Io et al., 2004). Prostaglandin E2 and MCP-1 enhance epithelial cell migration and induce the transcription of angiogenin-related genes, contributing to angiogenesis (Ito et al., 2000; Xiao et al., 2010). Moreover, peritoneal smooth muscle cells are activated after infection and induce the formation of new blood vessels by producing proangiogenic factors such as TGF-β, fibroblast growth factor, VEGF, tumour necrosis factor-α, and IL-8 (Aroeira et al., 2005). Inflammatory factors such as IL-1β and IL-6 may also participate in neovascularization by stimulating endothelial progenitor cell proliferation and inducing VEGF synthesis (Rosell et al., 2009; Catar et al., 2017).

## 7 Oxidative stress and glucose metabolism

Oxidative stress is a common occurrence in patients with chronic kidney disease (CKD) and its severity increases with the progression of the disease (Dounousi et al., 2006). In patients undergoing peritoneal dialysis, oxidative stress is mainly attributed to the bioincompatibility components of the peritoneal dialysis fluid, such as high glucose concentration, acidic pH, high osmolarity (Liakopoulos et al., 2017) and uraemic toxins (Castoldi et al., 2010). The accumulation of glucose degradation products (GDPs) in the peritoneum triggers the formation of excessive advanced glycation end products (AGEs), reactive oxygen species (ROS), and advanced oxidized protein products (AOPPs). The main pathway of ROS formation is through increased glucose oxidative metabolism triggered by GDPs and AGEs. The accumulation of AGEs in the peritoneal dialysis fluid promotes the expression and accumulation of specific multiligand transmembrane receptors, known as RAGE, in the peritoneum (Figure 1). These receptors induce morphological modifications of intracellular proteins, which alter the structure of the extracellular matrix (ECM) composition and the receptors expressed on the peritoneal cell membrane. Finally, ROS are produced when AGEs and AGE-modified proteins closely integrate with RAGE. Furthermore, ROS mediate the activation of proinflammatory factors, cytokines, transcription factors and growth factors, contributing to downstream aberrant gene transcription and apoptosis (Roumeliotis et al., 2020).

Si et al. found that PD fluid indeed induces metabolic reprogramming in the mouse peritoneum through conducting gene expression profiling and metabolomics analyses. Specifically, this reprogramming is characterized by a state of hyperglycolysis (Si et al., 2019). Correcting the metabolic state in mesothelial cells may be a therapeutic approach to treating peritoneal fibrosis (Si et al., 2019; Fu et al., 2022). GLUTs and SGLTs are involved in glucose transport and energy metabolism (Onishi et al., 2015; Han et al., 2022). The presence of high glucose levels in peritoneal dialysis fluid can potentially stimulate the expression of GLUT1 and SGLT1, leading to enhanced glucose absorption and a reduction of the osmotic gradient for ultrafiltration (Schröppel et al., 1998; Hong et al., 2016). Unfortunately, this process can ultimately contribute to the development of peritoneal fibrosis. With the continuous emergence of new hypoglycaemic drugs, some have found that SGLT-2 inhibitors reduce the absorption of glucose in peritoneal dialysate by inhibiting the activity of SGLT-2 (Zhou et al., 2019). They also significantly reduce the concentration of TGF-β, peritoneal thickening and fibrosis, and microvascular density, thus improving ultrafiltration (Balzer et al., 2020; Shentu et al., 2021). However, considering that the primary target of SGLT inhibitors in oral formulations is the renal tubules, it may be necessary to create more dosage forms, such as those that can be administered by intraperitoneal injection or added to peritoneal dialysis fluid, to improve peritoneal fibrosis.

## 8 Conclusion

In general, there are many factors involved in the development of peritoneal fibrosis in patients with abdominal dialysis, and the molecular signalling pathways involved are also complex, with interactions and influences on each other. Several factors are recognized for their importance in the development of peritoneal fibrosis in patients with PD. The most important is the use of traditional biocompatible peritoneal dialysis, which contains high glucose concentrations and glucose degradation products. Growth factor TGF-β1 is transformed by inducing many profibrotic events, including epithelium-mesenchymal transformation, fibroblast proliferation, and extracellular matrix deposition, playing a central role in peritoneal fibrosis. A solution containing high glucose stimulates the synthesis of TGF-β1 by activating protein kinase C in peritoneal cells and induces TGF-β type I and type II receptors, which induce various fibrosis-related signalling molecular transport pathways, including Smad-dependent and Smad-independent signalling pathways. Increasing studies on epigenetic regulation, hypoxic stimulation, and neovascularization are beginning to confirm their involvement. Peritonitis promotes peritoneal fibrosis through inflammatory factors such as IL-1β and IL-6, exacerbating the chronic induction of TGF-β1 synthesis (Kang et al., 1999), and promoting fibrosis events. Among the many factors involved in peritoneal fibrosis, EMT, the inflammatory response, autophagy, epigenetic regulation, and neovascularization are the dominant factors in fibrosis, of which TGF-β1 plays an important role in the activation of various signalling pathways, the induction of inflammatory factors, and the hypoxia stress response. Correspondingly, many scholars increasingly emphasize the proposed peritoneal mesothelial cell markers that reflect the inflammatory state of the peritoneum and the degree of fibrosis, and the application of clinical evaluation of peritoneal ultrafiltration function is an important part of the evaluation of the treatment effect. Carbohydrate antigen CA125, CTGF, suppression of tumorigenicity 2, MMP-2, and microRNAs (Mizutani et al., 2010; Ge et al., 2012; Barreto et al., 2013; Krediet and Struijk, 2013; Kim et al., 2019) are some of the biomarkers that have been proposed thus far, and the specificity and sensitivity of their diagnosis still need to be confirmed by more studies. Based on the above-related precipitating factors in the final treatment, the proposed peritoneal fibrosis treatments mainly include the use of biocompatible dialysate, tyrosine kinase inhibitors, inflammatory factor blockers, renin-angiotensin system inhibitors, and immunosuppressants, but most of these are still being studied in animal experiments or early clinical studies and are not widely used in clinical practice. In the future, more research is needed to further supplement the research targets and specific and detailed molecular mechanisms related to peritoneal fibrosis, and more clinical therapeutic drug intervention experiments will make greater contributions to delaying peritoneal fibrosis and providing more effective abdominal dialysis therapy for patients with PD.
